# *KIAA1109* gene mutation in surviving patients with Alkuraya-Kučinskas syndrome: a review of literature

**DOI:** 10.1186/s12881-020-01074-2

**Published:** 2020-06-26

**Authors:** Kishore Kumar, Anikha Bellad, Pramada Prasad, Satish Chandra Girimaji, Babylakshmi Muthusamy

**Affiliations:** 1 Institute of Bioinformatics, International Technology Park, Bangalore, 560066 India; 2 Manipal Academy of Higher Education, Manipal, Karnataka 576104 India; 3 Department of Neurology, NIMHANS, Hosur Road, Bangalore, 560029 India; 4 Department of Child and Adolescent Psychiatry, NIMHANS, Hosur Road, Bangalore, 560029 India

**Keywords:** Neonatal death, Premature termination of pregnancy, Prenatal diagnosis, KIAA clones, Mental retardation, Miscarriages, Developmental delay, Club foot, Arthrogryposis

## Abstract

**Background:**

Alkuraya-Kučinskas syndrome is an autosomal recessive disorder characterized by brain abnormalities associated with cerebral parenchymal underdevelopment, arthrogryposis, club foot and global developmental delay. *KIAA1109*, a functionally uncharacterized gene is identified as the molecular cause for Alkuraya-Kučinskas syndrome. Most of the reported mutations in *KIAA1109* gene result in premature termination of pregnancies or neonatal deaths while a few mutations have been reported in surviving patients with global developmental delay and intellectual disability. To our knowledge, only three surviving patients from two families have been reported with missense variants in *KIAA1109*. In this study, we describe four surviving patients from two related families (a multiplex family) with global developmental delay and mild to severe intellectual disability with no other systemic manifestations. There were no miscarriages or neonatal deaths reported in these families.

**Methods:**

X-chromosome exome panel sequencing was carried out in one patient and whole exome sequencing was carried out on the remaining three affected individuals and the unaffected father of the index family. Data analysis was carried out followed by variant filtering and segregation analysis. Sanger sequencing was carried out to validate the segregation of mutation in all four affected siblings and unaffected parents from both families.

**Results:**

A novel homozygous missense mutation in a conserved region of KIAA1109 protein was identified. Sanger sequencing confirmed the segregation of mutation in both families in an autosomal recessive fashion.

**Conclusion:**

Our study is the second study reporting a *KIAA1109* variant in surviving patients with Alkuraya-Kučinskas syndrome. Our study expands the spectrum of phenotypic features and mutations associated with Alkuraya-Kučinskas syndrome.

## Background

Alkuraya-Kučinskas syndrome (MIM: 617822) is an autosomal recessive disorder characterized by severe brain malformations, arthrogryposis and club foot. The brain abnormalities include cerebral parenchymal underdevelopment, lissencephaly, ventriculomegaly, reduced white matter volume, corpus callosum agenesis, dysplasia of cerebellum, hypoplasia of the pons and brainstem dysgenesis. Besides, abnormalities in multiple organ systems such as cardiac, renal and ophthalmologic were reported [1]. Loss of function mutations in *KIAA1109* have been reported as the cause for premature termination of pregnancies and perinatal deaths while some missense mutations were reported in surviving patients with global developmental delay and learning disabilities. The clinical features of the surviving patients with *KIAA1109* mutations are distinct as compared to the features observed in fetuses/new-borns carrying *KIAA1109* mutations that causes miscarriages and perinatal deaths. Surviving patients with *KIAA1109* mutations exhibited global developmental delay, mild to moderate learning disability, no development of speech, inability to stand or walk without support, muscle hypotonia, atrophy, stereotypic movements, dysmorphic features and early-onset epilepsy [1]. Behaviorally, the surviving patients exhibited poor concentration, self injuring behavior such as head-banging to express anger or frustration. Thus far, three surviving patients from two families have been reported with compound heterozygous missense variants [1].

In addition to Alkuraya-Kučinskas syndrome, *KIAA1109* has been reported as a significantly associated molecule in several autoimmune disorders including moderate to severe asthma [2], ulcerative colitis and celiac disease [3–5], rheumatoid arthritis [6], psoriatic arthritis [7], type 1 diabetes [8], anterior uveitis [9] and allergic sensitization [10]. It has also been identified to be associated with prostate cancer [11] and endometrial cancer [12].

In order to understand and characterize previously unidentified genes of larger size (> 50 KDa) in brain, Ohara et al and Kikuno et al cloned and sequenced 2031 cDNA clones from human brain library that includes 27 clones from fetal brain [13, 14]. The sequences of these genes are made publicly available in a repository known as Human Unidentified Gene-Encoded (HUGE) (http://www.kazusa.or.jp/huge/). As part of this project, *KIAA1109* gene was first sequenced from an adult human brain library that encodes a large protein product of 5005 amino acids. The GRCh38 annotation of *KIAA1109* gene in RefSeq lists one transcript (NM_015312.3) with experimental evidence and displays several other predicted transcripts including several non-protein coding transcripts of unknown significance [14]. *KIAA1109* is abundantly expressed in human ovary followed by the amygdala, cerebellum, subthalamic nucleus, thalamus and spinal cord with moderate to low expression in the hippocampus, caudate nucleus, corpus callosum, substantia nigra, fetal liver and fetal brain [14]. Low expression in fetal brain was detected at protein level as shown in the Human Proteome Map [15] (https://www.humanproteomemap.org/protein.php?hpm_id=84162). The GTEx portal shows ubiquitous expression of *KIAA1109* gene in 53 tissues that includes 13 different brain tissues (https://www.gtexportal.org/home/gene/KIAA1109). *KIAA1109* gene was discovered two decades ago, however, it remains uncharacterized. However, the published studies on this gene support its possible role in embryonic development [16] and the regulation of phagocytosis [17]. Tweek, the *Drosophila* ortholog of *KIAA1109* is involved in regulating synaptic vesicle recycling [18], thus suggesting its role in brain related functions.

In this study, we report four individuals from two related Indian families affected with intellectual disability and global developmental delay. Exome sequencing revealed a novel potentially pathogenic mutation in *KIAA1109* gene that well segregated in multiple members tested in this family. While several studies reported mutations in *KIAA1109* in fetal demises and neonatal deaths, this is the second study reporting a novel *KIAA1109* mutation in surviving patients with intellectual disability.

## Methods

In this study, we aimed at identifying the genetic cause of intellectual disability in four patients from two related families. Blood samples were collected from the parents and affected individuals. To identify the potentially pathogenic variants, we first carried out G-banded karyotyping in two patients, chromosome microarray analysis in two patients and exome sequencing in affected and unaffected members in the families. Sanger sequencing was performed to confirm the segregation of the potentially pathogenic variant identified in the family.

### Blood sample collection and karyotyping

Blood samples were collected from three affected male siblings from the index family and an affected male maternal cousin and the parents from both families. DNA isolation was carried out using QIAamp DNA minikit (Qiagen) as per the instrunctions given in the manufacturer’s protocol. G-banded karyotyping was first carried out on two affected individuals (F1.III-2 and F2.III-2) representing one patient in each family, as described previously [19].

### Chromosome microarray analysis

In order to identify larger genomic alterations, chromosomal microarray (CMA) analysis was performed on two patients (F1.IV-1 and F2.IV-2) representing one patient in each family, using a CytoScanTM 750 K array (Affymetrix, CA, USA). This microarray contains a comprehensive list of oligonucleotide probes (750 K) including a unique set of non-polymorphic probes (550 K) and a collection of bi-allelic single nucleotide polymorphism (SNP) probes (200 K) that enables genome wide screening of the variants. Analysis was carried out according to manufacturer’s protocol. Approximately, 250 ng of genomic DNA isolated from each of two affected individuals was digested with a restriction enzyme Nsp1. The digested fragments were then ligated with adapter sequences. These fragments were then PCR amplified using a single pair of primers that recognize the adapter sequences. Titanium Taq amplified PCR products of size 120 bp to 2000 bp were purified using AMP pure beads and fragmented to a range of product size of 25 bp to 125 bp. Subsequently, the fragmented PCR products were end labelled with biotin and the amplified and size selected products were then hybridized on CytoScan 750 K gene chip, and then scanned. Data analysis was performed using Chromosome Analysis Suite v1.2 (ChAS) (Affymetrix Inc., USA) based on human reference genome (GRCh37/hg19) using default parameters. The quality filtered raw data is subjected to CNV analysis. To identify the genes in CNVs and to evaluate the pathogenicity of the identified CNVs, we queried the following databases: i) Database of Genomic Variants (DGV) (http://dgv.tcag.ca/dgv/app/home), ii) DECIPHER (https://decipher.sanger.ac.uk/), iii) UCSC Genome browser (http://genome.ucsc.edu/) and iv) PubMed (http://www.ncbi.nlm.nih.gov/pubmed). In addition to the CNV analysis, loss of heterozygosity (LOH) analysis was also carried out using ChAS software.

### Exome sequencing

In order to identify single nucleotide variants and small insertions and deletions, we carried out exome sequencing on all affected indidivudals and an unaffected father (II-2). X-chromosome exome panel (X-panel) sequencing was carried out on the index patient and whole exome sequencing was carried out on the remaining three patients and unaffected father of index family. X-panel and whole exome sequencing and data analysis were carried out as described previously [19]. Briefly, paired end sequencing (2 × 150) reads were generated with a target depth of coverage of 100x. Quality filtered raw reads were aligned to the human reference genome (hg19) and post alignment quality recalibrated reads were subjected to joint variant calling across all five datasets. Variants were annotated using ANNOVAR [20] and rare variants with minor allele frequency < 0.01 were retained after comparing the variants with variants in 1000 genomes project [21], Exome Aggregation Consortium (ExAC) [22] and gnomAD (https://www.biorxiv.org/content/10.1101/531210v2). Subsequently, potential pathogenecity of the identified variants were evaluated using the publicly available tools such as SIFT [23], Polyphen-2 [24], MutationTaster-2 [25] and CADD [26]. Further, autosomal recessive variants, compound heterozygous variants and X-linked recessive variants were identified from filtered variants data. Compound heterozygous variants were identified using the following filtering steps: i) heterozygous variants in unaffected father and the same heterozygous variants in the patients, ii) a different heterozygous variant in the same gene found in patients but not present in the father which could have either been inherited from the mother or occurred as de novo mutations and iii) the potentially pathogenic nature of variants were interpreted manually.

### Sanger sequencing

Sanger sequencing was performed on the potentially pathogenic *KIAA1109* gene variant using the following procedure: Sanger confirmation was done on the index family (three affected siblings and their parents) and their cousin’s family (one affected individual and his parents). Primer Quest Tool (http://eu.idtdna.com/Primerquest/Home/Index) was used to design the primers (5′-TGCCAAGCAGCTATGTGTAAG-3′ and 5′-ATGTAAAGTCCTTGAACACCCA-3′) to amplify a 726 bp region of *KIAA1109.* OneTaq 2X Master Mix was used as per the manufacturer’s protocol. The DNA molecules were denatured at 95 °C for 30 s, followed by 35 cycles of denaturation at 95 °C for 30 s. The primers annealing temperature was set to 60 °C for 30 s and extended for 45 s at 68 °C. The final extension was at 68° for 5 min. The PCR products were cleaned and Sanger sequencing was carried out. The sequences obtained from sequencing were aligned to the reference genome to look for the mutations using BLAST tool. The chromatograms were analyzed manually to visualize the *KIAA1109* mutation to study its segregation in the patients and obligate carrier parents from both families.

## Results

### Patients

**Fig. 1:**
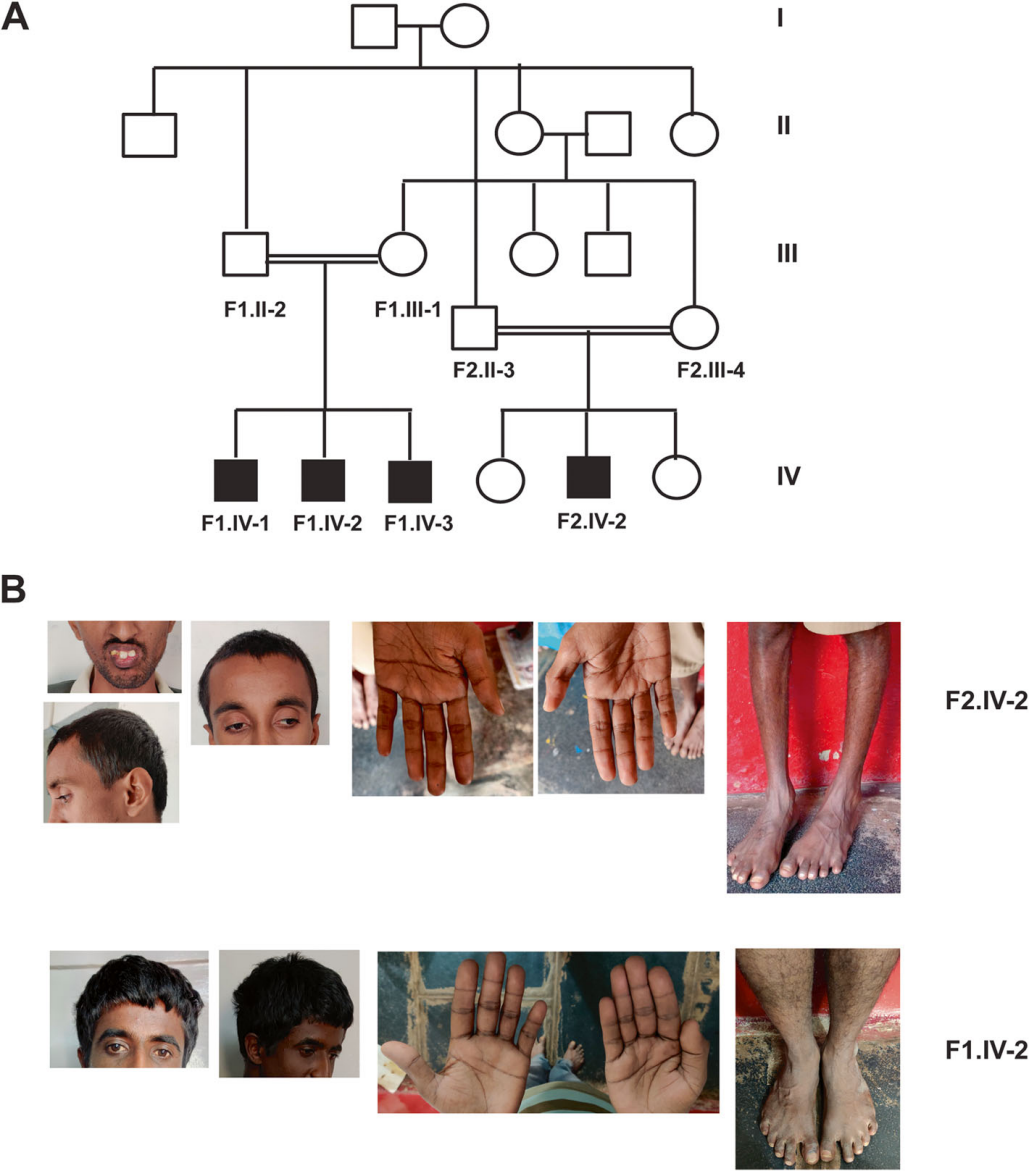
Pedigree and clinical photographs. **a** Pedigree depicting affected and unaffected individuals and consanguinity, **b** Clinical photographs describing dysmorphic features, simian crease and low muscle mass in legs of patient F2.IV-2 and F1.IV-2

The family described here is a multiplex family from the state of Karnataka in India. The multiplex family comprises four adult males affected with intellectual disability and global developmental delay. Two male siblings (F1.II-2 and F2.II-3) in this multiplex family had consanguineous marriages with the daughters (F1.III-1 and F2.III-4) of one of their sisters (II-4). The index family (F1) comprises three affected male siblings (F1.IV-1, F1.IV-2 and F1.IV-3). The second family (F2) comprises one affected male (F2.IV-2) and two unaffected females (Fig. 1a). The degree of intellectual disability varies among the affected siblings. The parents and the females in both families are phenotypically normal. The clinical photographs describing the phenotypic features of patients F1.IV-2 and F2.IV-2 representing each family is depicted in Fig. 1b**.**

All four boys were born by uncomplicated vaginal delivery following uneventful gestation. Patients F2.IV-2 and F1.IV-3 displayed delays in gross motor milestones with the former learning to walk independently only by 3 years, and the latter by about 18 months. These two patients also had delayed language milestones, learning to say two words by only about 5 and 7 years, respectively.

The four boys were brought to medical attention at the same time after the parents noticed F1.IV-1 underperform at school. They were brought to our clinic about 10 years ago when their ages were 28 (F1.IV-1), 21 (F1.IV-2), 14 (F2.IV-2), and 13 (F1.IV-3). They were diagnosed with intellectual disability (ID) of varying severity, with F2.IV-2 recording the lowest IQ of 29 as a case of severe ID. While F1.IV-3 was diagnosed with mild to moderate ID, it was also noted that he showed signs of social anxiety. F1-IV-1 was noted to show some dysmorphic features including a high arched palate with low set ears. None of the patients suffers from any other systemic illness.

Patients F1.IV-2 and F2.IV-2 were re-examined recently, however, the other two siblings declined to be assessed. F2.IV-2 at 24 years of age continues to have severe ID, with his speech restricted to phrases and short sentences. While he can read the clock and sign his name, he is unable to count beyond ten. His parents reported that he could care for himself, but being shy and asocial, he did not help with any outdoor errands. He also had a history of being impulsive and irritable with occasional headbanging. Though bashful during the examination he smiled when making eye contact. He had a lanky appearance with an elongated face, his arm span matching his height. His oral cavity showed a high-arched palate and protruding teeth on large gums. He had cubitus valgus and a palmar simian crease (right hand). Neurological examination revealed decreased muscle mass but was otherwise unremarkable. The rest of the systemic examination did not reveal anything significant.

At 31 years of age, F1.IV-2 is also intellectually disabled, though to a milder degree and he was more cooperative and amenable to being examined. He spoke clearly and coherently in short sentences. He was gainfully employed in the family business of weaving. Physical examination revealed a high arched palate, but the rest of the examination was unremarkable.

Table 1 describes the clinical findings from all four patients. A detailed comparison of phenotypic features of previously reported surviving patients and the phenotypes observed in these patients along with the identified/reported mutations are provided in Table 2. Unlike previously reported patients with *KIAA1109* mutations, there is no unexplained intrauterine fetal demise in these families. These patients did not have any overlapping features of patients reported previsouly with perinatal deaths.

**Table 1:** Clinical phenotypic features of all four patients

	F1.IV-1	F1.IV-2	F1.IV-3	F2.IV-2
**Age**	38	31	23	24
**Miscarriages/ neonatal deaths**	Nil	Nil	Nil	Nil
**Labour**	FTND	FTND	FTND	FTND
**Delayed Milestones – general**	Yes	Yes - mild	Yes - mild	Gross delay
**Walking independent**	1.5 years	1.5 years	1.5 years	3 years
**Speaking two meaningful words**	1.5 years	1.5 years	6–7 years	4–5 years
**Other salient historical points**	NS	NS	NS	Brain imaging done ~ 5 y back, reportedly abnormal, records not available
**ID severity**	Mild	Mild	Moderate with expressive speech delay	severe
**IQ**	ND	57	43	29
**Behavior**	Shy, uncooperative	Cooperative, self-care fair	Social anxiety	Impulsive, irritable, short tempered, stubborn, occasional head-banging, shy/asocial
**Height/cm**	163	161	Could not be measured	164 Arm-span (164)
**Head Circumference/cm**	54	55	42	55.5
**Chest Circumference/cm**	Could not be measured	82	Could not be measured	72
**Mid-arm Circumference/cm**	Could not be measured	24	Could not be measured	18
**Vitals**	Could not be measured	BP 124/80 mmHg	Could not be measured	BP 100/60 mmHg
**Major Congenital Anomalies**	Nil	Nil	Nil	Nil
**Minor Congenital Anomalies**	High arched palate, low set ears, prominent eyebrows, curly hair, mild beaking of the nose	High arched palate	Could not be examined	Malformed dentition, high arched palate, pointed ear on rt. side, long face, simian crease rt. hand, long thumb, small sub-mental region, cubitus valgus, high-arched foot
**External genitalia**	Normal	Could not be examined	Could not be examined	normal
**Neurological Examination, salient findings**	No apparent abnormalities	No apparent abnormalities	No apparent abnormalities	Muscle bulk less, rest of the systemic examination. No apparent abnormalities

Patients F1.IV-2 and F2.IV-2 were examined on 20 Dec 2019. The physical examination findings of the other two patients are from the archives. None of the patients has a history of seizures or other systemic illnesses. *NS* Nothing significant, *ND* Not done

**Table 2:** Mutations and phenotypic features of surviving patients with KIAA1109 mutations

Reference	This study, F1.IV-1	This study, F1.IV-2	This study, F1.IV-3	This study, F2.IV-2	Gueneau, et al., 2018 [1]	Gueneau, et al., 2018 [1]	Gueneau, et al., 2018 [1]
**Sex, Age**	Male, 38 yo	Male, 31 yo	Male, 23 yo	Male, 24 yo	Male, 13 yo	Female, 7 yo	Female, 11 yo
**Mutation**	g. 123140678A > G; c.2431A > G (hom)	g. 123140678A > G; c.2431A > G (hom)	g. 123140678A > G; c.2431A > G (hom)	g. 123140678A > G; c.2431A > G (hom)	Chr4:123160823; c.3986A > C (het) Chr4:123170727; c.5599G > A (het)	Chr4:123160823; c.3986A > C (het) ^a^Chr4:123170727; c.5599G > A (het)	Chr4:123164200; c.4719G > A (het) and Chr4:123171679; c.5873G > A (het)
**Protein Change**	p.Thr811Ala	p.Thr811Ala	p.Thr811Ala	p.Thr811Ala	p.Tyr1329Cys and p.Val1867Met	p.Tyr1329Cys and p.Val1867Met	p.Met1573Ile and p.Arg1958Gln
**ID**	Mild	Mild	Moderate	Severe	Severe	Severe	Moderate
**Neuropsychiatric**	Delayed motor milestones, shy and uncooperative	Delayed motor milestones	Expressive speech delay, delayed motor milestones, social anxiety	Delayed motor and language milestones, impulsive, irritable, short-tempered, stubborn, occasional head-banging, shy/asocial	Global developmental delay, no language, cannot stand or walk without support, early onset epilepsy	Global developmental delay, no language, cannot sit or stand without support, stereotypic movements, early onset epilepsy	Global developmental delay, mild to moderate learning disability, poor concentration, immature behavior with minor self-harm (head-banging) when angry/frustrated
**Imaging**	Not done	Not done	Not done	Not available	Post-natal brain MRI: small posterior fossa arachnoid cyst, discrete vermian atrophy, slight increase in the fluid-filled retro- and infracerebellar space and mild enlargement of subarachnoid spaces of frontal regions	Post-natal brain MRI: discrete parenchymal rarefaction involving the frontal lobes	Prenatal imaging (US and MRI): major microcephaly (HC −5 SD) with reduced white matter volume and mild ventriculomegaly
**Head and neck**	High arched palate, low set ears, prominent eyebrows, mild beaking of the nose	High arched palate	–	Malformed dentition, high arched palate, pointed ear on right side, long face, simian crease right hand, long thumb, small sub-mental region	Plagiocephaly, refractive errors of the eyes, delayed dentition	Plagiocephaly, refractive errors of the eye, strabismus	Hypertelorism, slightly upslanting palpebral fissures, ocular motor apraxia, refractive errors of the eye, strabismus, dental crowding, high palate
**Skeletal System**	–	–	–	Cubitus valgus, high-arched foot, reduced muscle bulk	Mild contractures of large joints, syndactyly of 2nd and 3rd toes, limb paresis at birth, talipes valgus, muscle hypotonia and atrophy	Mild contractures of large joints, paretic position of hands and feet in infancy, talipes valgus, muscle hypotonia and atrophy	Asymmetry of the thorax, mild bilateral talipes, syndactyly of 2nd and 3rd toes, 5th toe clinodacytly, hallux valgus
**GI**	–	–	–	–	–	Chronic constipation	Gastroesophageal reflux
**Heart**	–	–	–	–	–	–	Tetralogy of Fallot with pulmonary atresia
**Urogenital**	–	–	–	–	Scrotal hypoplasia	–	–
**Other**	–	–	–	–	–	Dermatitis, psoriasis	–

^a^The genomic nucleotide mentioned by Gueneau et al., 2018 [1] seems to be wrong

### Karyotyping and chromosome microarray analysis

In order to look for any chromosomal abnormalities, G-banded karyotyping was carried out on two affected siblings F1.VI-2 and F2.IV-2 representing one patient in each family. The chromosomes were normal with 46, XY complement. Since the patients exhibited non-syndromic phenotypes including developmental delay and intellectual disability, the diagnostic yield of G-banded karyotyping is ~ 3% and chromosome microarray analysis is often recommended as a first-tier diagnostics test [27]. CytoScan 750 K Array was used since it has a comprehensive list of probes with higher sensitivity and specificity to detect copy number variations in two affected individuals representing one in each family. Karyotypes were checked first which revealed normal chromosomes with 46, XY complement, consistent with G-banded karyotyping. CNV analysis was carried out using ChAS software and identified with CNVs and LOH regions. CNVs devoid of gene content or reported in general population were removed and the clinical relevance of the the identified CNVs were evaluated manually. The CNVs identified in both the patients were compared and we found that there is no potentially pathogenic CNVs addressing the phenotypes observed in the patients. Similarly, LOH analysis also did not yield any significant clinically relevant variants.

### Exome sequencing and identification of a novel mutation in KIAA1109

Based on the inference from the pedigree, because only males are affected, we initially carried out X-panel sequencing of the index patient (F1.IV-1) expecting X-linked recessive inheritance. Data analysis revealed no significant X-linked variants that fit the phenotypes observed in the patients. Whole exome sequencing was then carried out on the remaining patients (F1.IV-2, F1.IV-3 and F2.IV-2) and unaffected father (F1.II-2) of the index family (F1). Joint variant calling identified a total of 1,613,810 unfiltered variants. Exonic, and splice site variants were retained and common variants with minor allele frequency < 0.01 were removed. A list of qualifying rare variants are provided in Supplementary Table **1**. After applying autosomal recessive mode of inheritance pattern, a novel variant g.123140678A > G was identified that was found to be a homozygous in all three affected individuals (F1.IV-2, F1.IV-3 and F2.IV-2) and heterozygous in the unaffected father (F1.II-2). This variant is located at 4q27, a locus of autoimmune disorders [28]. This mutation was found in exon 19 of *KIAA1109* gene (c.2431A > G) resulting in a missense variant altering threonine to alanine at position 811, p.Thr811Ala in KIAA1109 protein (RefSeq: NM_015312.3 (transcript) and NP_056127.2 (protein)) (Table 2). *KIAA1109* encodes for a large protein that has no known functional domains or motifs. Although *KIAA1109* gene is discovered two decades ago, it remains functionally uncharacterized. This variant is not reported in dbSNP, 1000 genomes project, ExAC and gnomAD databases. Polyphen-2 predicted the effect of p.Thr811Ala mutation as probably damaging (Score: 0.994), SIFT predicted the effect of the mutation as damaging (score: 0.05). MutationTaster predicted the effect of the mutation as disease-causing (score: 1).

**Fig. 2:**
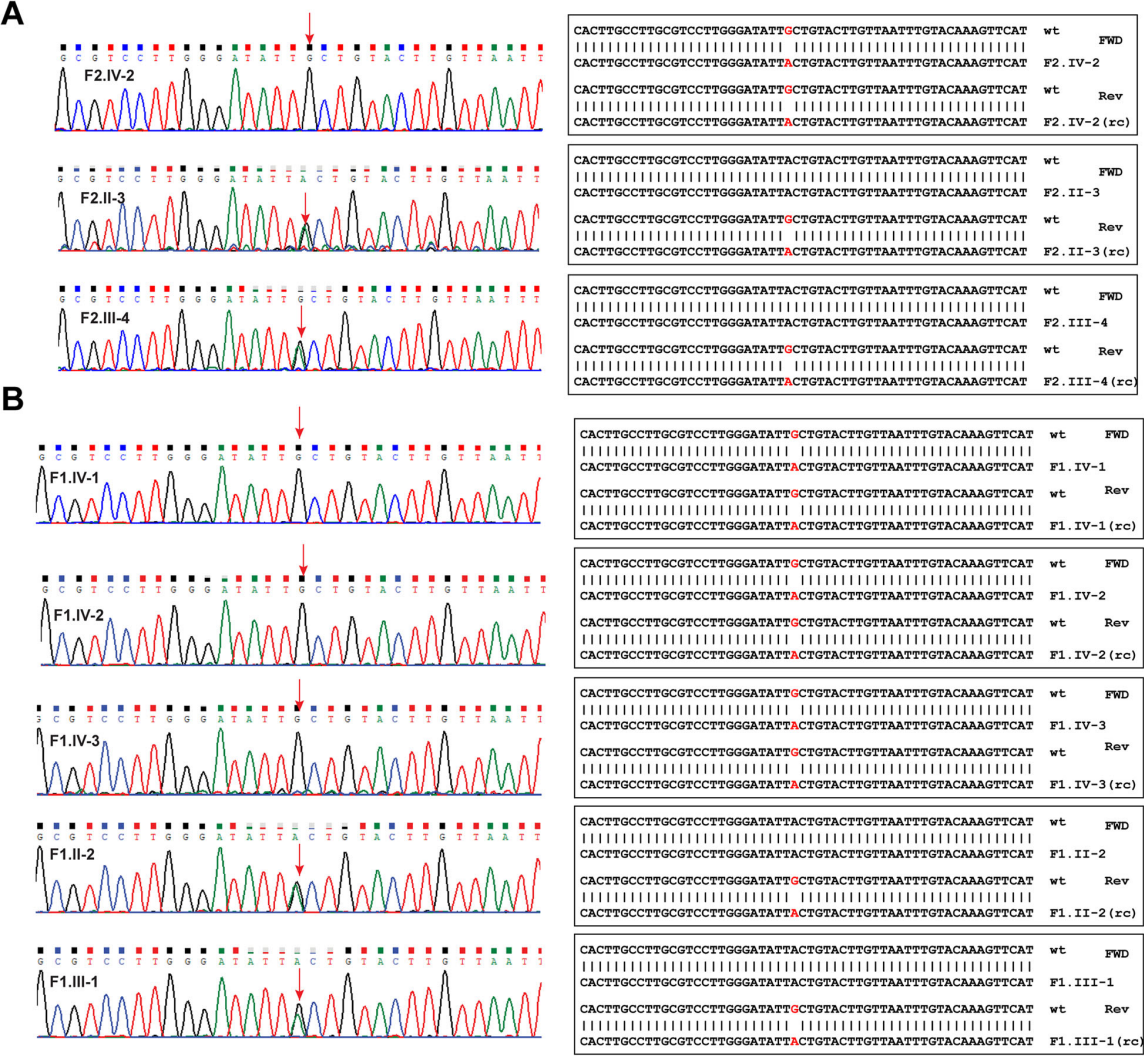
Sanger validation of *KIAA1109* mutation in patients and their parents. Figure depicting chromatogram, and sequence alignments of Sanger results of *KIAA1109* mutation g.123140678A > G of **a** F2.IV-2 and his parents and **b** patients F1.IV-1, F1.IV-2 and F1.IV-2 and their parents (index family). The mutated nucleotides are shown by red arrow (in chromatogram) or marked red (in sequence alignment). Sequence alignments are shown for both forward and reverse strands sequences to depict the homozygosity (in patients) or heterozygosity (in parents). Reverse strand sequences were reverse complemented before alignment

**Fig. 3:**
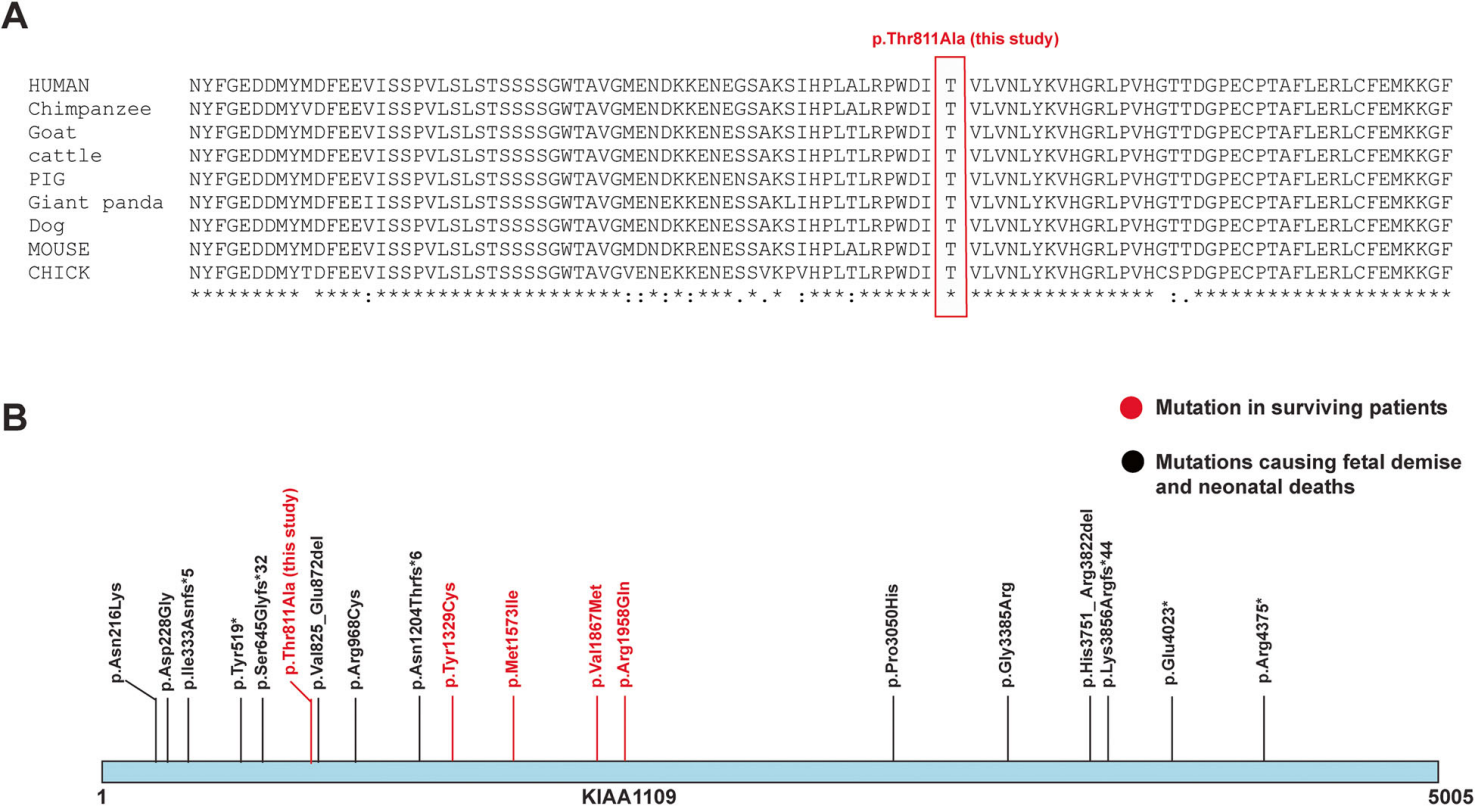
Conservation analysis and protein altering mutations of KIAA1109. **a** conservation analysis showing the conservation of p.Thre811Ala mutation of KIAA1109 identified in this study across species. **b** Depiction of known mutations and novel mutation identified in this study causing Alkuraya-Kucinskas syndrome. Mutations shown in red are the mutations in surviving patients and those shown in black are the mutations identified in patients with fetal demises and neonatal deaths

In order to validate the *KIAA1109* variant g.123140678A > G and to study the segregation of mutation in the family, we carried out Sanger sequencing on the three affected individuals in the index family (F1.IV-1, F1.IV-2, F1.IV-3), their parents (F1.II-3, F1.III-1), affected cousin (F2.IV-2) and his parents (F2.II-3, F2.III-4). The chromatograms of Sanger results confirmed the homozygous mutation in all affected patients and heterozygous mutation in all unaffected parents confirming the segregation of mutation in the family (Fig. 2a **&** Fig. 2b). Conservation analysis of a region spanning p.Thr811Ala across species shows the conservation of the mutated residue and the residues around the site of the mutation (Fig. 3a).

## Discussion

KIAA1109 is a large and evolutionarily conserved protein with no known domains or motifs. A number of mutations in *KIAA1109* have been reported in miscarriages and/or neonatal deaths and only one study has reported surviving patients with *KIAA1109* mutations (Fig. 3b).

### *KIAA1109* mutations causing miscarriages and neonatal deaths

The association of *KIAA1109* with neurogenetic disorders was first reported from a single family of a large cohort study of 143 consanguineous families comprising of two female patients (deceased) with Dandy-Walker malformation, hydrocephalus, flexed deformity, club feet, micrognathia, and pleural effusion [29]. Another study identified a large homozygous deletion spanning exons 28 to 55 in four neonatal deaths with patients exhibiting severe arthrogryposis and axillar pterygium [30]. The reason for death and age of death were not indicated. A subsequent study screening 44 families with terminating pregnancies identified two unrelated families with *KIAA1109* mutations: i) a truncating mutation was reported in a patient with hydrocephalus, hypoplastic cerebellum, skin edema and bilateral talipes with a history of two preceding intrauterine fetal deaths at 6 months of pregnancy with similar presentations (severe hydrocephalus, spina bifida, and polyhydramnios) and ii) a splice site mutation was identified in a patient with hydrocephalus and arthrogryposis [16]. Subsequently, the same group published 13 patients from 10 families with *KIAA1109* mutations in fetuses with miscarriages and neonatal deaths. In addition to these, this study has reported three surviving patients with global developmental delay [1]. In a Russian family with a history of two miscarriages, two intronic compound heterozygous variants were identified both altering splicing leading to premature termination of the translation with a deletion of 46 amino acids of KIAA1109 protein [31]. These fetuses exhibited bilateral ventriculomegaly, arthrogryposis, bilateral pyelectasis, increased thickness of the nuchal fold and hypoplastic and low set ears and prenatal diagnosis aided in choosing the right pregnancy [31]. Subsequently, six patients with death during the neonatal period have been reported with similar overlapping phenotypes as described in previous patients with *KIAA1109* mutations [32]. The growing literature on *KIAA1109* mutations in premature termination of pregnancies demonstrates its possible role in development [31]. These studies indicate that *KIAA1109* plays a crucial role in fetal development. Although fetal malformations can be detected in utero using advanced imaging techniques, molecular diagnosis provides additional clues for a more accurate diagnosis.

### *KIAA1109* mutations in surviving patients with intellectual disability and developmental delay

Interestingly, only one previous study has reported missense mutations in *KIAA1109* in surviving patients in a Lithuanian family with a male and a female children of ages 13 and 7 years, respectively exhibiting severe developmental delay and no speech **(**Table 2**)** [1]. Another British family with an 11 years old child exhibiting global developmental delay and mild to moderate learning disabilities has been reported (Table 2) [1]. We report here the third family with *KIAA1109* mutation in surviving patients with global developmental delay and intellectual disability (Table 2). Unlike the other surviving cases reported, none of the patients in this study had major congenital anomalies or musculoskeletal involvement such as congenital heart disease, arthrogryposis, ocular manifestations or dysmorphic features. Thus, compared to surviving cases reported earlier, these patients had much milder phenotypes with varying degrees of ID as the predominant feature that expands the genotype and phenotype spectrum of Alkuraya-Kučinskas Syndrome.

## Conclusions

Our description of the four surviving patients significantly expands the phenotypic features of Alkuraya-Kučinskas Syndrome. Our study is the second study to provide evidence for a role of *KIAA1109* in intellectual disability in surviving patients. Identification of such mutations differentiating the mutations responsible for fetal demise and intellectual disability will greatly enhance the accuracy of prenatal diagnosis to aid informed decision making by prospective parents. A high degree of precision is essential for accurate genetic counseling and the pursuit of preventive options such as preimplantation and prenatal diagnosis. Molecular experiments to understand and differentiate the regions of KIAA1109 protein in manifesting these groups of disorders are warranted.

## Supplementary information

**Additional file 1: Supplementary Table S1.** List of qualitative rare variants identified from four patients and unaffected father of index family.

## Data Availability

The datasets generated and analysed during the current study are made available as a supplementary table (Supplementary Table **1**) that lists all qualified variants. Human Proteome Map: https://www.humanproteomemap.org/protein.php?hpm_id=84162; GTEx portal: https://www.gtexportal.org/home/gene/KIAA1109; Human genome GRCh37: https://ftp.ncbi.nlm.nih.gov/genomes/all/GCF/000/001/405/GCF_000001405.39_GRCh38.p13/GCF_000001405.39_GRCh38.p13_genomic.fna.gz; Population frequency annotation and functional effect prediction were annotated through ANNOVAR: https://doc-openbio.readthedocs.io/projects/annovar/en/latest/user-guide/filter/ 1000 genomes project, phase 3 ID: 1000g2015aug; ExAC ID: exac03; gnomAD exome ID: gnomad_exome; gnomAD genome ID: gnomad_genome; Scores of SIFT, Polyphen, MutationTaster and CADD from ANNOVAR ID: dbnsfp30a.

## Abbreviations

MIM: Mendelian inheritance in man
cDNA: Complementary deoxyribonucleic acid
HUGE: Human unidentified gene-encoded
GRCh38: Genome reference consortium human build 38
BWA: Burrows-Wheeler aligner
PCR: Polymerase chain reaction
GVCF: Genomic variant call format
GATK: Genome analysis toolkit
ExAC: Exome aggregation consortium
gnomAD: Genome aggregation database
CADD: Combined annotation dependent depletion
CMA: Chromosome microarray
SNP: Single nucleotide polymorphism
ChAS: Chromosome analysis suite
CNV: Copy number variation
DGV: Database of genomic variants
LOH: Loss of heterozygosity
UCSC: University of california santa cruz
BLAST: Basic local alignment search tool
ID: Intellectual disability
